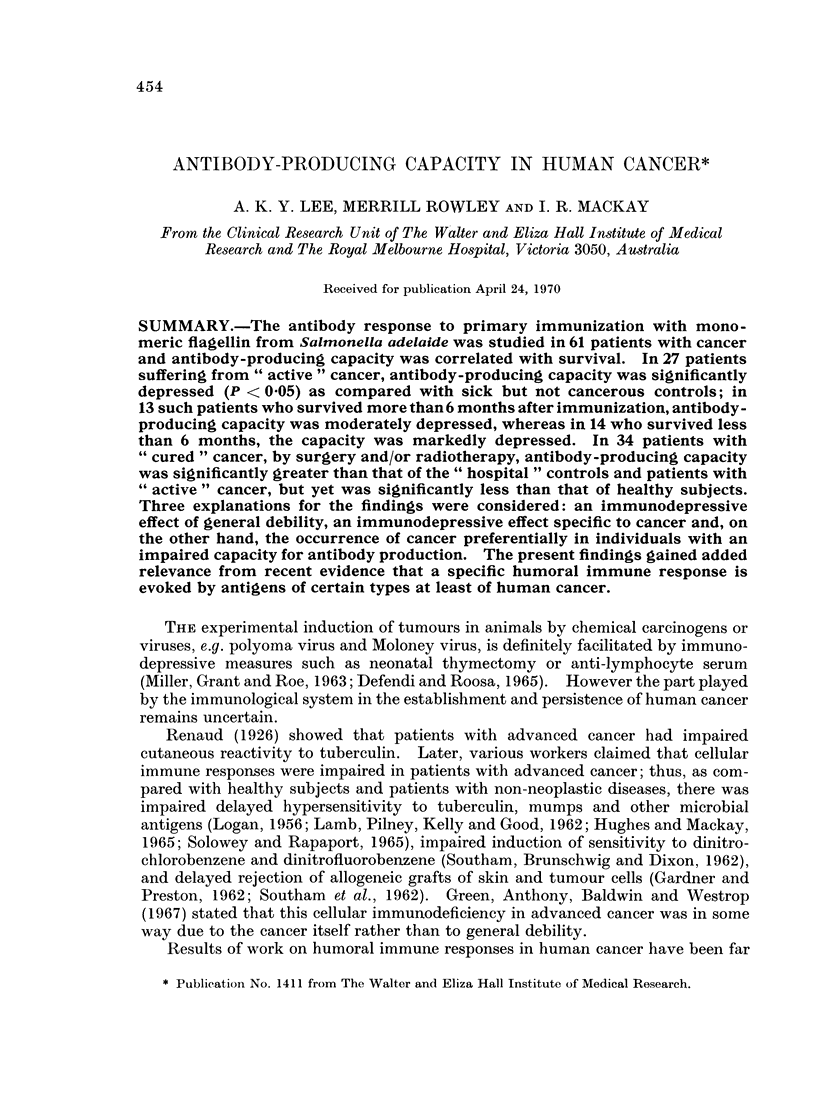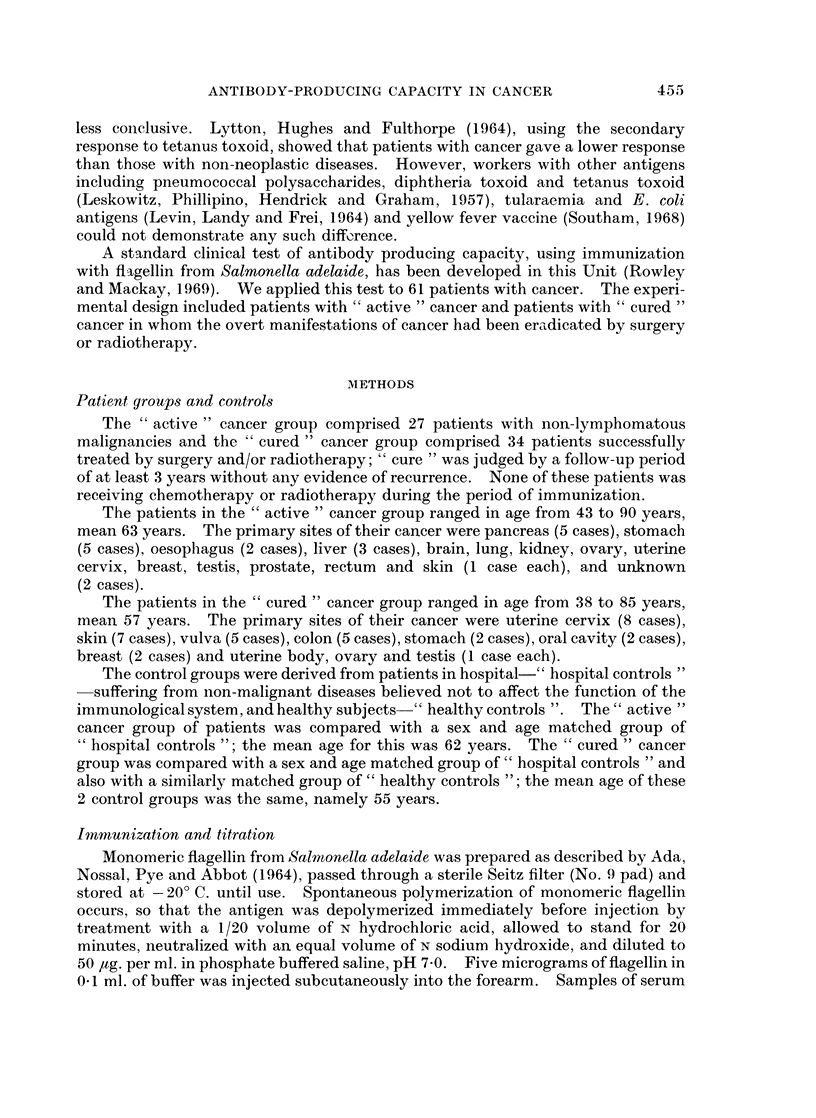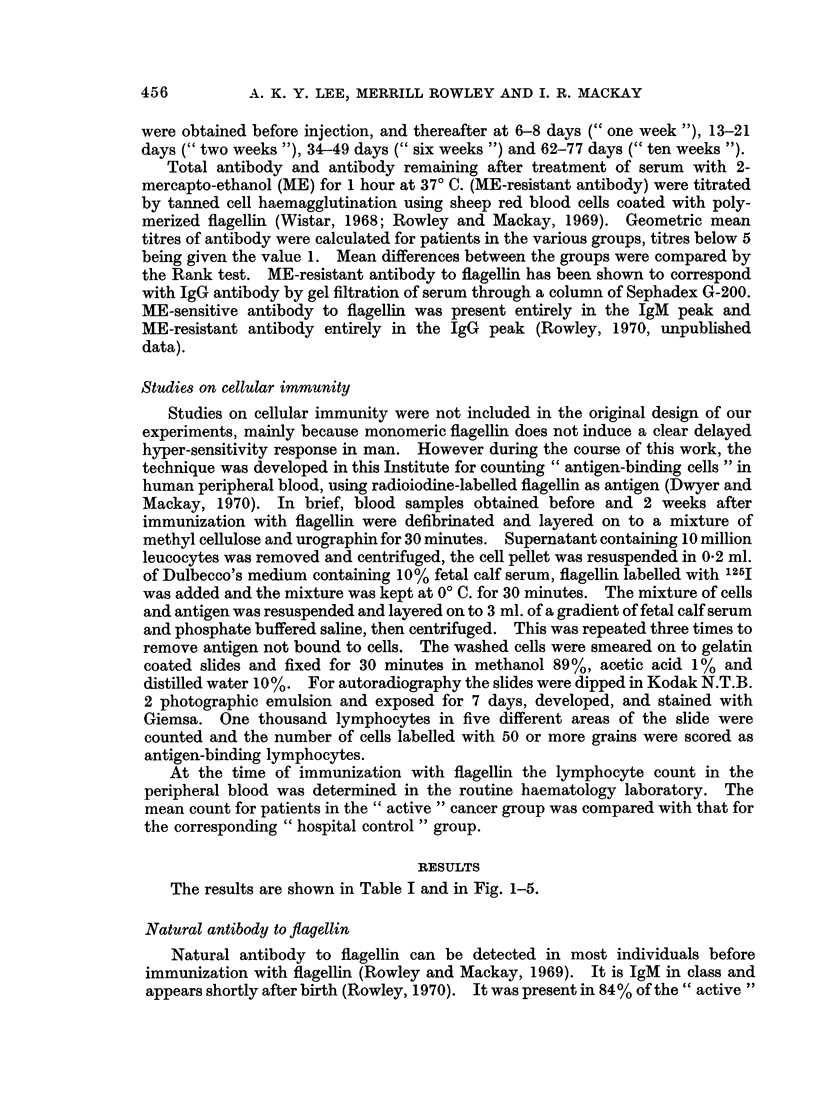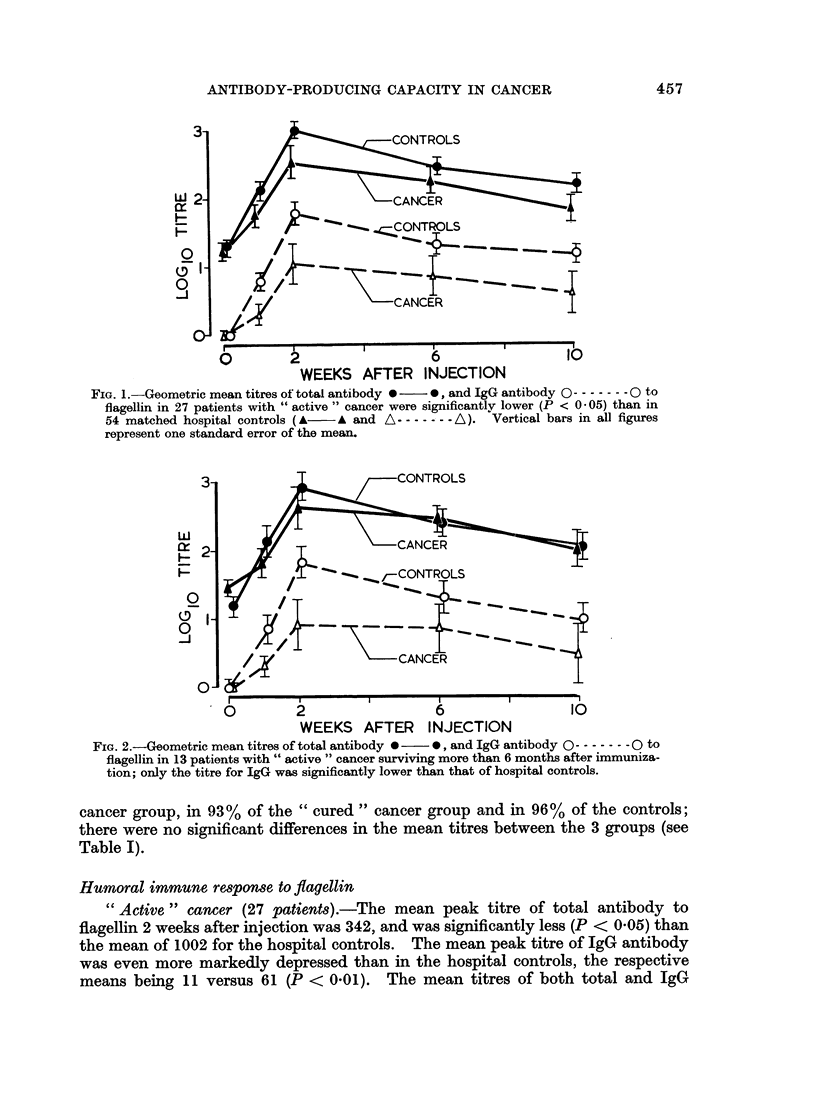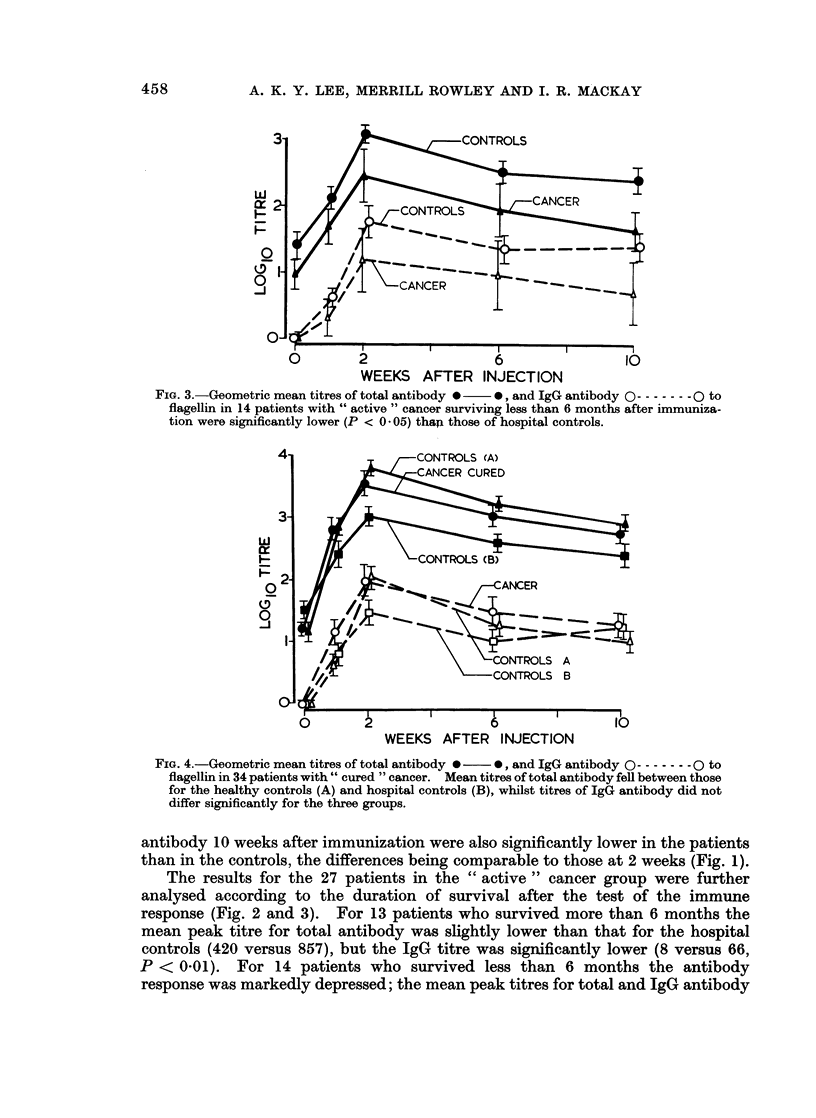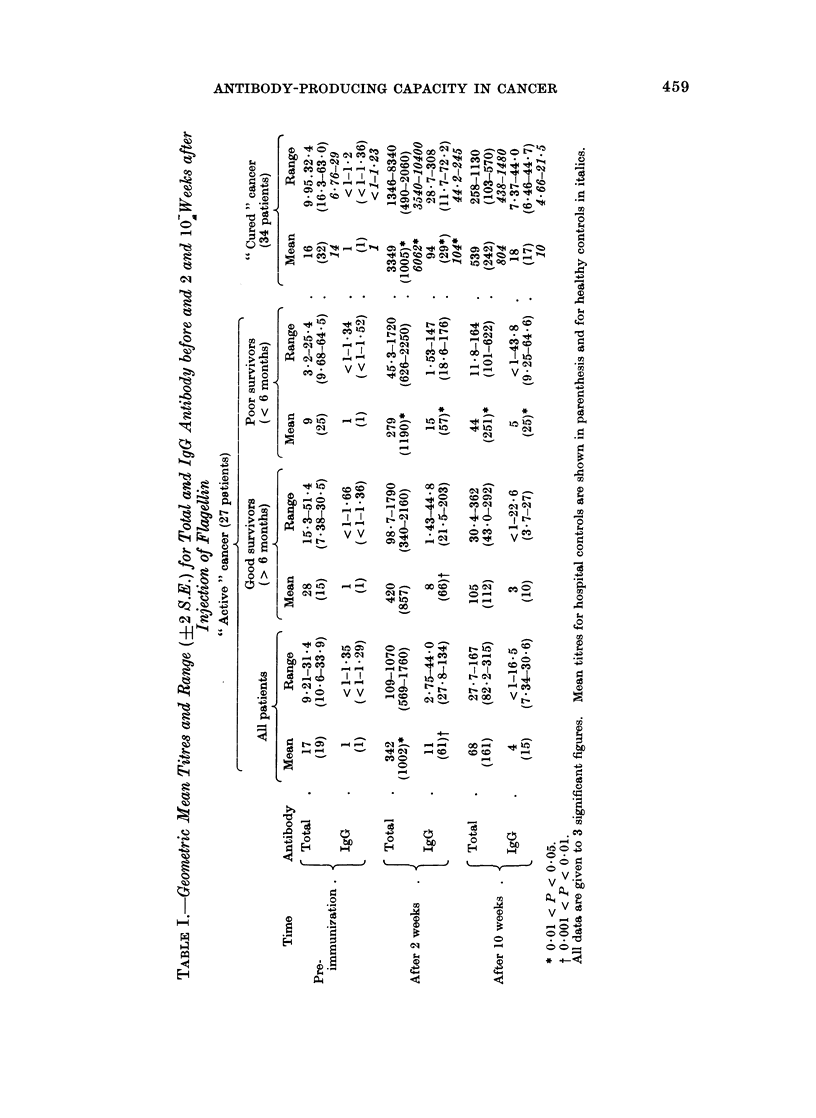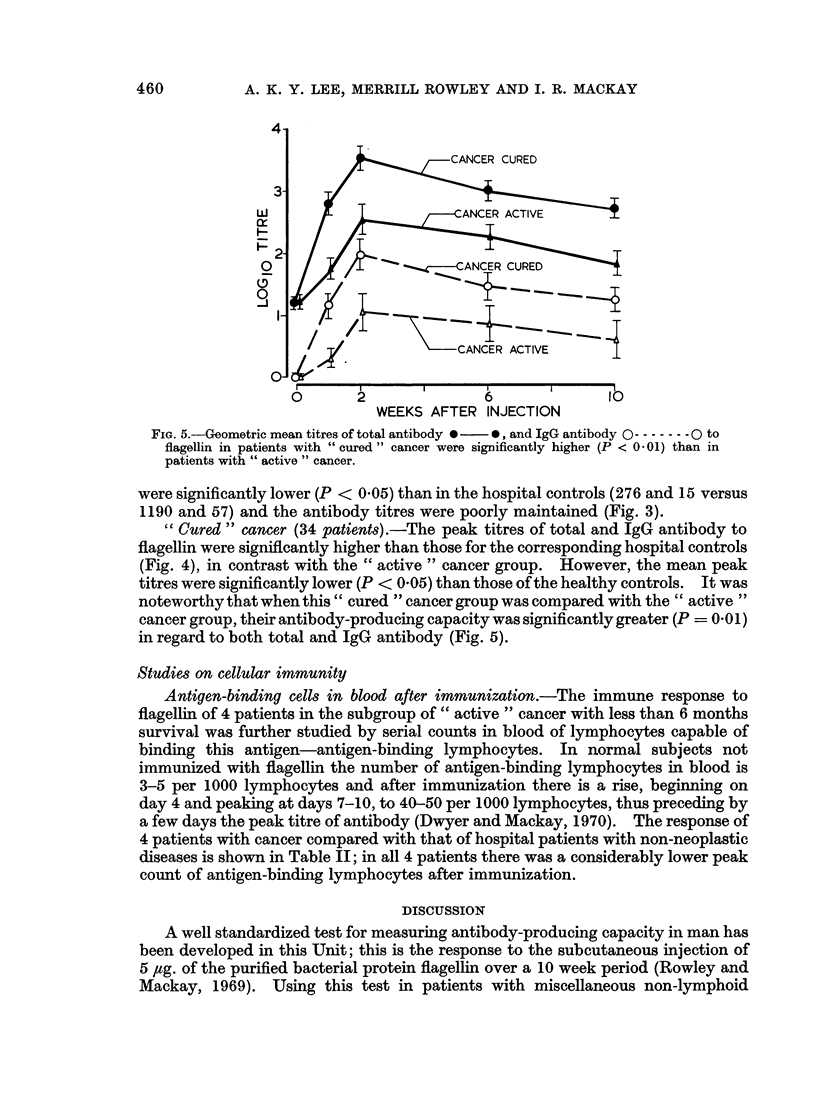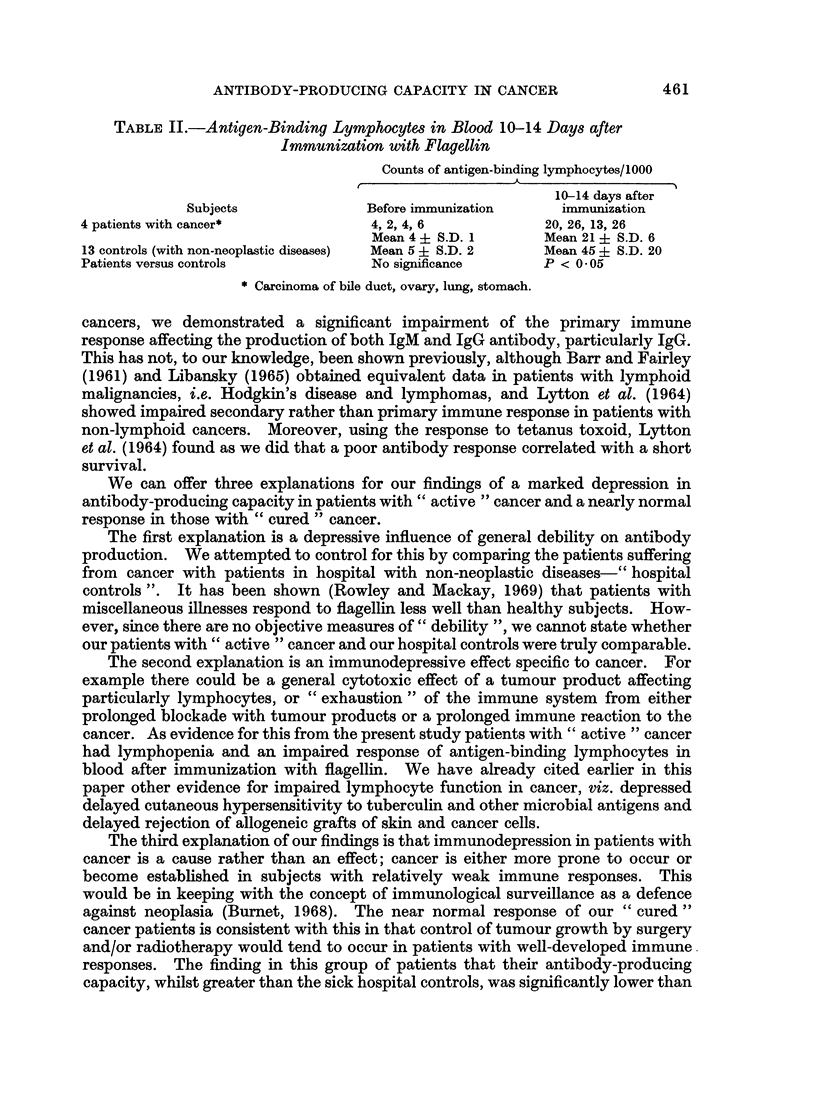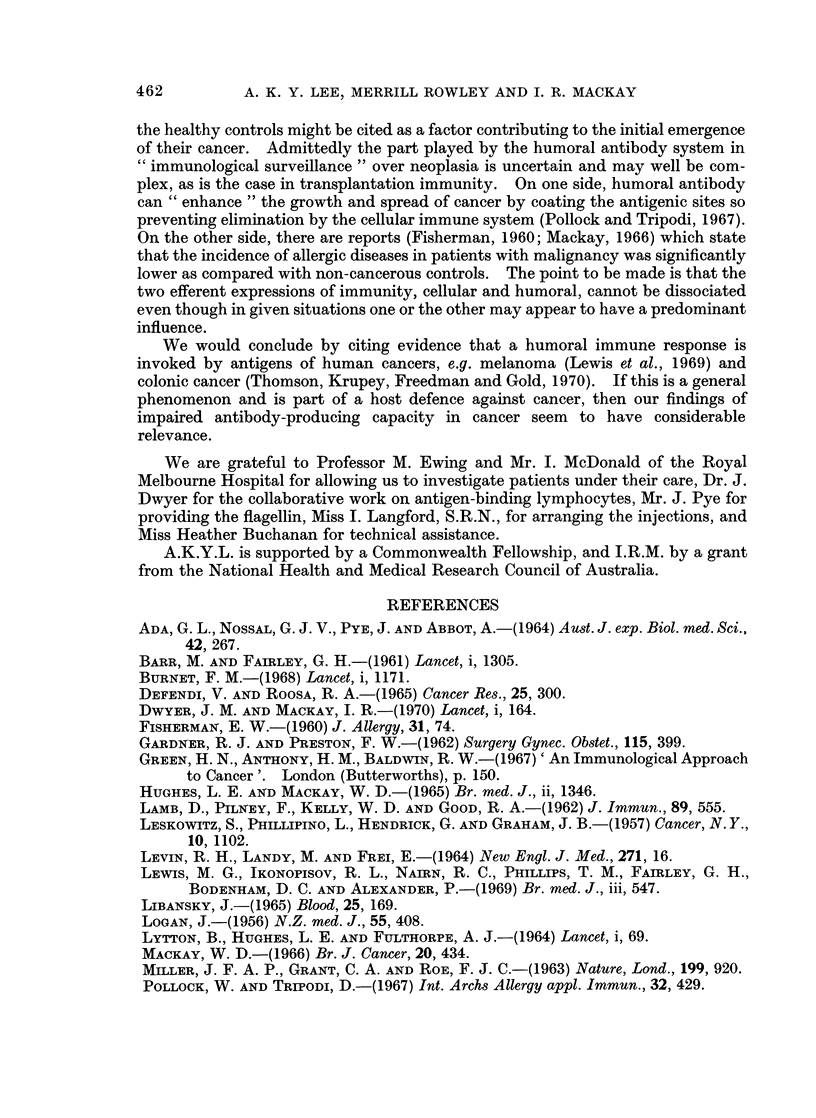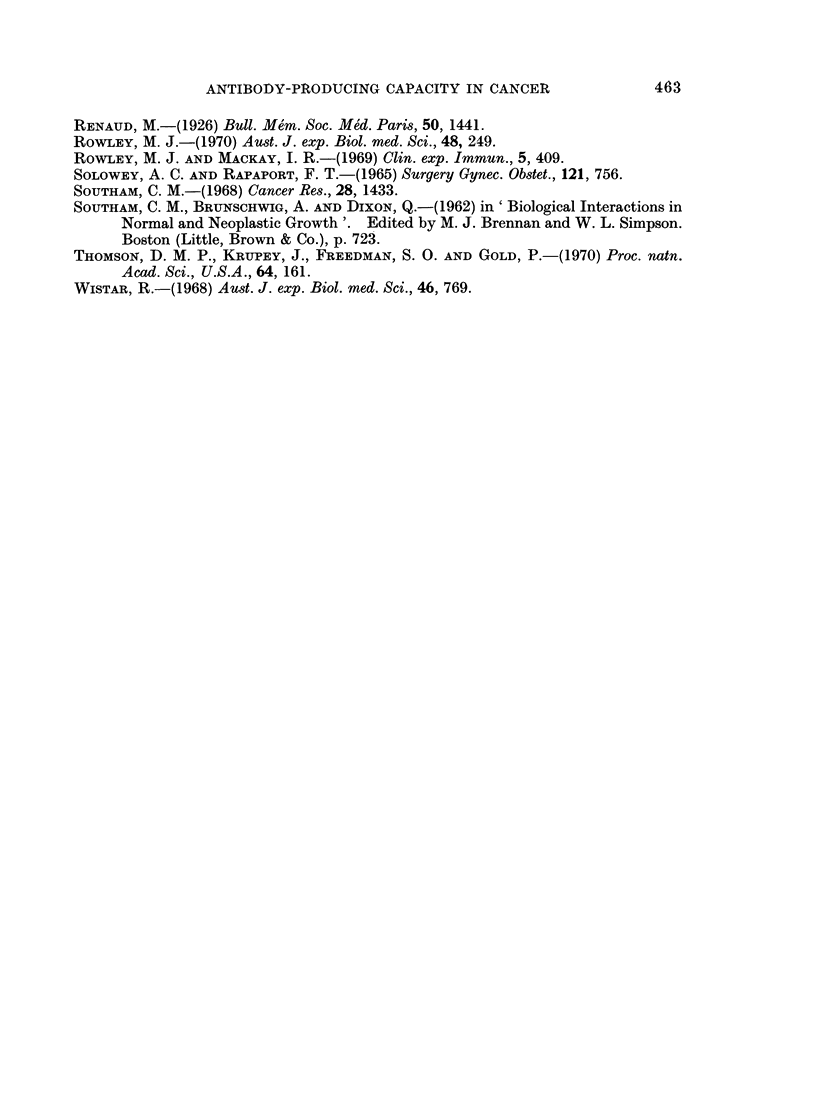# Antibody-producing Capacity in Human Cancer*

**DOI:** 10.1038/bjc.1970.54

**Published:** 1970-09

**Authors:** A. K. Y. Lee, Merrill Rowley, I. R. Mackay

## Abstract

The antibody response to primary immunization with monomeric flagellin from *Salmonella adelaide* was studied in 61 patients with cancer and antibody-producing capacity was correlated with survival. In 27 patients suffering from “active” cancer, antibody-producing capacity was significantly depressed (*P*<0.05) as compared with sick but not cancerous controls; in 13 such patients who survived more than 6 months after immunization, antibody-producing capacity was moderately depressed, whereas in 14 who survived less than 6 months, the capacity was markedly depressed. In 34 patients with “cured” cancer, by surgery and/or radiotherapy, antibody-producing capacity was significantly greater than that of the “hospital” controls and patients with “active” cancer, but yet was significantly less than that of healthy subjects. Three explanations for the findings were considered: an immunodepressive effect of general debility, an immunodepressive effect specific to cancer and, on the other hand, the occurrence of cancer preferentially in individuals with an impaired capacity for antibody production. The present findings gained added relevance from recent evidence that a specific humoral immune response is evoked by antigens of certain types at least of human cancer.


### ANTIBODY-PRODUCING CAPACITY IN HUMAN CANCER*

A. K. Y. LEE, MERRILL ROWLEY AND I. R. MACKAY

*From the Clinical Research Unit of The Walter and Eliza Hall Institute of Medical Research and The Royal Melbourne Hospital, Victoria* 3050, *Australia*

Received for publication April 24, 1970

**SUMMARY.—The antibody response to primary immunization with monomeric flagellin from *Salmonella adelaide* was studied in 61 patients with cancer and antibody-producing capacity was correlated with survival. In 27 patients suffering from " active " cancer, antibody-producing capacity was significantly depressed ($P < 0{\cdot}05$) as compared with sick but not cancerous controls; in 13 such patients who survived more than 6 months after immunization, antibody-producing capacity was moderately depressed, whereas in 14 who survived less than 6 months, the capacity was markedly depressed. In 34 patients with " cured " cancer, by surgery and/or radiotherapy, antibody-producing capacity was significantly greater than that of the " hospital " controls and patients with " active " cancer, but yet was significantly less than that of healthy subjects. Three explanations for the findings were considered: an immunodepressive effect of general debility, an immunodepressive effect specific to cancer and, on the other hand, the occurrence of cancer preferentially in individuals with an impaired capacity for antibody production. The present findings gained added relevance from recent evidence that a specific humoral immune response is evoked by antigens of certain types at least of human cancer.**

THE experimental induction of tumours in animals by chemical carcinogens or viruses, *e.g.* polyoma virus and Moloney virus, is definitely facilitated by immunodepressive measures such as neonatal thymectomy or anti-lymphocyte serum (Miller, Grant and Roe, 1963; Defendi and Roosa, 1965). However the part played by the immunological system in the establishment and persistence of human cancer remains uncertain.

Renaud (1926) showed that patients with advanced cancer had impaired cutaneous reactivity to tuberculin. Later, various workers claimed that cellular immune responses were impaired in patients with advanced cancer; thus, as compared with healthy subjects and patients with non-neoplastic diseases, there was impaired delayed hypersensitivity to tuberculin, mumps and other microbial antigens (Logan, 1956; Lamb, Pilney, Kelly and Good, 1962; Hughes and Mackay, 1965; Solowey and Rapaport, 1965), impaired induction of sensitivity to dinitrochlorobenzene and dinitrofluorobenzene (Southam, Brunschwig and Dixon, 1962), and delayed rejection of allogeneic grafts of skin and tumour cells (Gardner and Preston, 1962; Southam *et al.*, 1962). Green, Anthony, Baldwin and Westrop (1967) stated that this cellular immunodeficiency in advanced cancer was in some way due to the cancer itself rather than to general debility.

Results of work on humoral immune responses in human cancer have been far

* Publication No. 1411 from The Walter and Eliza Hall Institute of Medical Research.

less conclusive. Lytton, Hughes and Fulthorpe (1964), using the secondary response to tetanus toxoid, showed that patients with cancer gave a lower response than those with non-neoplastic diseases. However, workers with other antigens including pneumococcal polysaccharides, diphtheria toxoid and tetanus toxoid (Leskowitz, Phillipino, Hendrick and Graham, 1957), tularaemia and *E. coli* antigens (Levin, Landy and Frei, 1964) and yellow fever vaccine (Southam, 1968) could not demonstrate any difference.

A standard clinical test of antibody producing capacity, using immunization with flagellin from *Salmonella adelaide*, has been developed in this Unit (Rowley and Mackay, 1969). We applied this test to 61 patients with cancer. The experimental design included patients with " active " cancer and patients with " cured " cancer in whom the overt manifestations of cancer had been eradicated by surgery or radiotherapy.

### METHODS

### *Patient groups and controls*

The " active " cancer group comprised 27 patients with non-lymphomatous malignancies and the " cured " cancer group comprised 34 patients successfully treated by surgery and/or radiotherapy; " cure " was judged by a follow-up period of at least 3 years without any evidence of recurrence. None of these patients was receiving chemotherapy or radiotherapy during the period of immunization.

The patients in the " active " cancer group ranged in age from 43 to 90 years, mean 63 years. The primary sites of their cancer were pancreas (5 cases), stomach (5 cases), oesophagus (2 cases), liver (3 cases), brain, lung, kidney, ovary, uterine cervix, breast, testis, prostate, rectum and skin (1 case each), and unknown (2 cases).

The patients in the " cured " cancer group ranged in age from 38 to 85 years, mean 57 years. The primary sites of their cancer were uterine cervix (8 cases), skin (7 cases), vulva (5 cases), colon (5 cases), stomach (2 cases), oral cavity (2 cases), breast (2 cases) and uterine body, ovary and testis (1 case each).

The control groups were derived from patients in hospital—" hospital controls " —suffering from non-malignant diseases believed not to affect the function of the immunological system, and healthy subjects—" healthy controls ". The " active " cancer group of patients was compared with a sex and age matched group of " hospital controls "; the mean age for this was 62 years. The " cured " cancer group was compared with a sex and age matched group of " hospital controls " and also with a similarly matched group of " healthy controls "; the mean age of these 2 control groups was the same, namely 55 years.

### *Immunization and titration*

Monomeric flagellin from *Salmonella adelaide* was prepared as described by Ada, Nossal, Pye and Abbot (1964), passed through a sterile Seitz filter (No. 9 pad) and stored at $-20°$ C. until use. Spontaneous polymerization of monomeric flagellin occurs, so that the antigen was depolymerized immediately before injection by treatment with a 1/20 volume of N hydrochloric acid, allowed to stand for 20 minutes, neutralized with an equal volume of N sodium hydroxide, and diluted to 50 $\mu$g. per ml. in phosphate buffered saline, pH 7·0. Five micrograms of flagellin in 0·1 ml. of buffer was injected subcutaneously into the forearm. Samples of serum were obtained before injection, and thereafter at 6–8 days (" one week "), 13–21 days (" two weeks "), 34–49 days (" six weeks ") and 62–77 days (" ten weeks ").

Total antibody and antibody remaining after treatment of serum with 2-mercapto-ethanol (ME) for 1 hour at 37° C. (ME-resistant antibody) were titrated by tanned cell haemagglutination using sheep red blood cells coated with polymerized flagellin (Wistar, 1968; Rowley and Mackay, 1969). Geometric mean titres of antibody were calculated for patients in the various groups, titres below 5 being given the value 1. Mean differences between the groups were compared by the Rank test. ME-resistant antibody to flagellin has been shown to correspond with IgG antibody by gel filtration of serum through a column of Sephadex G-200. ME-sensitive antibody to flagellin was present entirely in the IgM peak and ME-resistant antibody entirely in the IgG peak (Rowley, 1970, unpublished data).

*Studies on cellular immunity*

Studies on cellular immunity were not included in the original design of our experiments, mainly because monomeric flagellin does not induce a clear delayed hyper-sensitivity response in man. However during the course of this work, the technique was developed in this Institute for counting " antigen-binding cells " in human peripheral blood, using radioiodine-labelled flagellin as antigen (Dwyer and Mackay, 1970). In brief, blood samples obtained before and 2 weeks after immunization with flagellin were defibrinated and layered on to a mixture of methyl cellulose and urographin for 30 minutes. Supernatant containing 10 million leucocytes was removed and centrifuged, the cell pellet was resuspended in 0·2 ml. of Dulbecco's medium containing 10% fetal calf serum, flagellin labelled with $^{125}I$ was added and the mixture was kept at 0° C. for 30 minutes. The mixture of cells and antigen was resuspended and layered on to 3 ml. of a gradient of fetal calf serum and phosphate buffered saline, then centrifuged. This was repeated three times to remove antigen not bound to cells. The washed cells were smeared on to gelatin coated slides and fixed for 30 minutes in methanol 89%, acetic acid 1% and distilled water 10%. For autoradiography the slides were dipped in Kodak N.T.B. 2 photographic emulsion and exposed for 7 days, developed, and stained with Giemsa. One thousand lymphocytes in five different areas of the slide were counted and the number of cells labelled with 50 or more grains were scored as antigen-binding lymphocytes.

At the time of immunization with flagellin the lymphocyte count in the peripheral blood was determined in the routine haematology laboratory. The mean count for patients in the " active " cancer group was compared with that for the corresponding " hospital control " group.

### RESULTS

The results are shown in Table I and in Fig. 1–5.

*Natural antibody to flagellin*

Natural antibody to flagellin can be detected in most individuals before immunization with flagellin (Rowley and Mackay, 1969). It is IgM in class and appears shortly after birth (Rowley, 1970). It was present in 84% of the " active "

FIG. 1.—Geometric mean titres of total antibody ●——●, and IgG antibody ○- - - - - - -○ to flagellin in 27 patients with " active " cancer were significantly lower ($P < 0 \cdot 05$) than in 54 matched hospital controls (▲——▲ and △- - - - - - -△). Vertical bars in all figures represent one standard error of the mean.

FIG. 2.—Geometric mean titres of total antibody ●——●, and IgG antibody ○- - - - - - -○ to flagellin in 13 patients with " active " cancer surviving more than 6 months after immunization; only the titre for IgG was significantly lower than that of hospital controls.

cancer group, in 93% of the " cured " cancer group and in 96% of the controls; there were no significant differences in the mean titres between the 3 groups (see Table I).

*Humoral immune response to flagellin*

" *Active* " *cancer* (27 *patients*).—The mean peak titre of total antibody to flagellin 2 weeks after injection was 342, and was significantly less ($P < 0 \cdot 05$) than the mean of 1002 for the hospital controls. The mean peak titre of IgG antibody was even more markedly depressed than in the hospital controls, the respective means being 11 versus 61 ($P < 0 \cdot 01$). The mean titres of both total and IgG

FIG. 3.—Geometric mean titres of total antibody ●——●, and IgG antibody ○- - - - - - -○ to flagellin in 14 patients with " active " cancer surviving less than 6 months after immunization were significantly lower ($P < 0{\cdot}05$) than those of hospital controls.

FIG. 4.—Geometric mean titres of total antibody ●——●, and IgG antibody ○- - - - - - -○ to flagellin in 34 patients with " cured " cancer. Mean titres of total antibody fell between those for the healthy controls (A) and hospital controls (B), whilst titres of IgG antibody did not differ significantly for the three groups.

antibody 10 weeks after immunization were also significantly lower in the patients than in the controls, the differences being comparable to those at 2 weeks (Fig. 1).

The results for the 27 patients in the " active " cancer group were further analysed according to the duration of survival after the test of the immune response (Fig. 2 and 3). For 13 patients who survived more than 6 months the mean peak titre for total antibody was slightly lower than that for the hospital controls (420 versus 857), but the IgG titre was significantly lower (8 versus 66, $P < 0{\cdot}01$). For 14 patients who survived less than 6 months the antibody response was markedly depressed; the mean peak titres for total and IgG antibody

TABLE I.—*Geometric Mean Titres and Range (±2 S.E.) for Total and IgG Antibody before and 2 and 10 Weeks after Injection of Flagellin*

| Time | Antibody | "Active" cancer (27 patients): All patients — Mean | All patients — Range | Good survivors (> 6 months) — Mean | Good survivors (> 6 months) — Range | Poor survivors (< 6 months) — Mean | Poor survivors (< 6 months) — Range | "Cured" cancer (34 patients) — Mean | "Cured" cancer (34 patients) — Range |
|---|---|---|---|---|---|---|---|---|---|
| Pre-immunization | Total | 17 | 9·21–31·4 | 28 | 15·3–51·4 | 9 | 3·2–25·4 | 16 | 9·95.32·4 |
| | | (19) | (10·6–33·9) | (15) | (7·38–30·5) | (25) | (9·68–64·5) | (32) | (16·3–63·0) |
| | | | | | | | | *14* | *6·76–29* |
| | IgG | 1 | < 1–1·35 | 1 | < 1–1·66 | 1 | < 1–1·34 | 1 | < 1–1·2 |
| | | (1) | (< 1–1·29) | (1) | (< 1–1·36) | (1) | (< 1–1·52) | (1) | (< 1–1·36) |
| | | | | | | | | *1* | *< 1–1·23* |
| After 2 weeks | Total | 342 | 109–1070 | 420 | 98·7–1790 | 279 | 45·3–1720 | 3349 | 1346–8340 |
| | | (1002)* | (569–1760) | (857) | (340–2160) | (1190)* | (626–2250) | (1005)* | (490–2060) |
| | | | | | | | | *6062** | *3540–10400* |
| | IgG | 11 | 2·75–44·0 | 8 | 1·43–44·8 | 15 | 1·53–147 | 94 | 28·7–308 |
| | | (61)† | (27·8–134) | (66)† | (21·5–203) | (57)* | (18·6–176) | (29*) | (11·7–72·2) |
| | | | | | | | | *104** | *44·2–245* |
| After 10 weeks | Total | 68 | 27·7–167 | 105 | 30·4–362 | 44 | 11·8–164 | 539 | 258–1130 |
| | | (161) | (82·2–315) | (112) | (43·0–292) | (251)* | (101–622) | (242) | (103–570) |
| | | | | | | | | *804* | *438–1480* |
| | IgG | 4 | < 1–16·5 | 3 | < 1–22·6 | 5 | < 1–43·8 | 18 | 7·37–44·0 |
| | | (15) | (7·34–30·6) | (10) | (3·7–27) | (25)* | (9·25–64·6) | (17) | (6·46–44·7) |
| | | | | | | | | *10* | *4·66–21·5* |

* $0 \cdot 01 < P < 0 \cdot 05$.

† $0 \cdot 001 < P < 0 \cdot 01$.

All data are given to 3 significant figures. Mean titres for hospital controls are shown in parenthesis and for healthy controls in italics.

FIG. 5.—Geometric mean titres of total antibody ●——●, and IgG antibody ○- - - - - - -○ to flagellin in patients with " cured " cancer were significantly higher ($P < 0 \cdot 01$) than in patients with " active " cancer.

were significantly lower ($P < 0 \cdot 05$) than in the hospital controls (276 and 15 versus 1190 and 57) and the antibody titres were poorly maintained (Fig. 3).

" *Cured* " *cancer* (34 *patients*).—The peak titres of total and IgG antibody to flagellin were significantly higher than those for the corresponding hospital controls (Fig. 4), in contrast with the " active " cancer group. However, the mean peak titres were significantly lower ($P < 0 \cdot 05$) than those of the healthy controls. It was noteworthy that when this " cured " cancer group was compared with the " active " cancer group, their antibody-producing capacity was significantly greater ($P = 0 \cdot 01$) in regard to both total and IgG antibody (Fig. 5).

### *Studies on cellular immunity*

*Antigen-binding cells in blood after immunization.*—The immune response to flagellin of 4 patients in the subgroup of " active " cancer with less than 6 months survival was further studied by serial counts in blood of lymphocytes capable of binding this antigen—antigen-binding lymphocytes. In normal subjects not immunized with flagellin the number of antigen-binding lymphocytes in blood is 3–5 per 1000 lymphocytes and after immunization there is a rise, beginning on day 4 and peaking at days 7–10, to 40–50 per 1000 lymphocytes, thus preceding by a few days the peak titre of antibody (Dwyer and Mackay, 1970). The response of 4 patients with cancer compared with that of hospital patients with non-neoplastic diseases is shown in Table II; in all 4 patients there was a considerably lower peak count of antigen-binding lymphocytes after immunization.

### DISCUSSION

A well standardized test for measuring antibody-producing capacity in man has been developed in this Unit; this is the response to the subcutaneous injection of 5 $\mu$g. of the purified bacterial protein flagellin over a 10 week period (Rowley and Mackay, 1969). Using this test in patients with miscellaneous non-lymphoid

TABLE II.—*Antigen-Binding Lymphocytes in Blood* 10–14 *Days after Immunization with Flagellin*

| Subjects | Counts of antigen-binding lymphocytes/1000: Before immunization | 10–14 days after immunization |
|---|---|---|
| 4 patients with cancer* | 4, 2, 4, 6 | 20, 26, 13, 26 |
| | Mean 4 ± S.D. 1 | Mean 21 ± S.D. 6 |
| 13 controls (with non-neoplastic diseases) | Mean 5 ± S.D. 2 | Mean 45 ± S.D. 20 |
| Patients versus controls | No significance | $P < 0 \cdot 05$ |

* Carcinoma of bile duct, ovary, lung, stomach.

cancers, we demonstrated a significant impairment of the primary immune response affecting the production of both IgM and IgG antibody, particularly IgG. This has not, to our knowledge, been shown previously, although Barr and Fairley (1961) and Libansky (1965) obtained equivalent data in patients with lymphoid malignancies, *i.e.* Hodgkin's disease and lymphomas, and Lytton *et al.* (1964) showed impaired secondary rather than primary immune response in patients with non-lymphoid cancers. Moreover, using the response to tetanus toxoid, Lytton *et al.* (1964) found as we did that a poor antibody response correlated with a short survival.

We can offer three explanations for our findings of a marked depression in antibody-producing capacity in patients with " active " cancer and a nearly normal response in those with " cured " cancer.

The first explanation is a depressive influence of general debility on antibody production. We attempted to control for this by comparing the patients suffering from cancer with patients in hospital with non-neoplastic diseases—" hospital controls ". It has been shown (Rowley and Mackay, 1969) that patients with miscellaneous illnesses respond to flagellin less well than healthy subjects. However, since there are no objective measures of " debility ", we cannot state whether our patients with " active " cancer and our hospital controls were truly comparable.

The second explanation is an immunodepressive effect specific to cancer. For example there could be a general cytotoxic effect of a tumour product affecting particularly lymphocytes, or " exhaustion " of the immune system from either prolonged blockade with tumour products or a prolonged immune reaction to the cancer. As evidence for this from the present study patients with " active " cancer had lymphopenia and an impaired response of antigen-binding lymphocytes in blood after immunization with flagellin. We have already cited earlier in this paper other evidence for impaired lymphocyte function in cancer, *viz.* depressed delayed cutaneous hypersensitivity to tuberculin and other microbial antigens and delayed rejection of allogeneic grafts of skin and cancer cells.

The third explanation of our findings is that immunodepression in patients with cancer is a cause rather than an effect; cancer is either more prone to occur or become established in subjects with relatively weak immune responses. This would be in keeping with the concept of immunological surveillance as a defence against neoplasia (Burnet, 1968). The near normal response of our " cured " cancer patients is consistent with this in that control of tumour growth by surgery and/or radiotherapy would tend to occur in patients with well-developed immune responses. The finding in this group of patients that their antibody-producing capacity, whilst greater than the sick hospital controls, was significantly lower than the healthy controls might be cited as a factor contributing to the initial emergence of their cancer. Admittedly the part played by the humoral antibody system in " immunological surveillance " over neoplasia is uncertain and may well be complex, as is the case in transplantation immunity. On one side, humoral antibody can " enhance " the growth and spread of cancer by coating the antigenic sites so preventing elimination by the cellular immune system (Pollock and Tripodi, 1967). On the other side, there are reports (Fisherman, 1960; Mackay, 1966) which state that the incidence of allergic diseases in patients with malignancy was significantly lower as compared with non-cancerous controls. The point to be made is that the two efferent expressions of immunity, cellular and humoral, cannot be dissociated even though in given situations one or the other may appear to have a predominant influence.

We would conclude by citing evidence that a humoral immune response is invoked by antigens of human cancers, *e.g.* melanoma (Lewis *et al.*, 1969) and colonic cancer (Thomson, Krupey, Freedman and Gold, 1970). If this is a general phenomenon and is part of a host defence against cancer, then our findings of impaired antibody-producing capacity in cancer seem to have considerable relevance.

We are grateful to Professor M. Ewing and Mr. I. McDonald of the Royal Melbourne Hospital for allowing us to investigate patients under their care, Dr. J. Dwyer for the collaborative work on antigen-binding lymphocytes, Mr. J. Pye for providing the flagellin, Miss I. Langford, S.R.N., for arranging the injections, and Miss Heather Buchanan for technical assistance.

A.K.Y.L. is supported by a Commonwealth Fellowship, and I.R.M. by a grant from the National Health and Medical Research Council of Australia.

### REFERENCES

ADA, G. L., NOSSAL, G. J. V., PYE, J. AND ABBOT, A.—(1964) *Aust. J. exp. Biol. med. Sci.*, **42**, 267.

BARR, M. AND FAIRLEY, G. H.—(1961) *Lancet*, i, 1305.

BURNET, F. M.—(1968) *Lancet*, i, 1171.

DEFENDI, V. AND ROOSA, R. A.—(1965) *Cancer Res.*, **25**, 300.

DWYER, J. M. AND MACKAY, I. R.—(1970) *Lancet*, i, 164.

FISHERMAN, E. W.—(1960) *J. Allergy*, **31**, 74.

GARDNER, R. J. AND PRESTON, F. W.—(1962) *Surgery Gynec. Obstet.*, **115**, 399.

GREEN, H. N., ANTHONY, H. M., BALDWIN, R. W.—(1967) ' An Immunological Approach to Cancer '. London (Butterworths), p. 150.

HUGHES, L. E. AND MACKAY, W. D.—(1965) *Br. med. J.*, ii, 1346.

LAMB, D., PILNEY, F., KELLY, W. D. AND GOOD, R. A.—(1962) *J. Immun.*, **89**, 555.

LESKOWITZ, S., PHILLIPINO, L., HENDRICK, G. AND GRAHAM, J. B.—(1957) *Cancer, N.Y.*, **10**, 1102.

LEVIN, R. H., LANDY, M. AND FREI, E.—(1964) *New Engl. J. Med.*, **271**, 16.

LEWIS, M. G., IKONOPISOV, R. L., NAIRN, R. C., PHILLIPS, T. M., FAIRLEY, G. H., BODENHAM, D. C. AND ALEXANDER, P.—(1969) *Br. med. J.*, iii, 547.

LIBANSKY, J.—(1965) *Blood*, **25**, 169.

LOGAN, J.—(1956) *N.Z. med. J.*, **55**, 408.

LYTTON, B., HUGHES, L. E. AND FULTHORPE, A. J.—(1964) *Lancet*, i, 69.

MACKAY, W. D.—(1966) *Br. J. Cancer*, **20**, 434.

MILLER, J. F. A. P., GRANT, C. A. AND ROE, F. J. C.—(1963) *Nature, Lond.*, **199**, 920.

POLLOCK, W. AND TRIPODI, D.—(1967) *Int. Archs Allergy appl. Immun.*, **32**, 429.

RENAUD, M.—(1926) *Bull. Mém. Soc. Méd. Paris*, **50**, 1441.
ROWLEY, M. J.—(1970) *Aust. J. exp. Biol. med. Sci.*, **48**, 249.
ROWLEY, M. J. AND MACKAY, I. R.—(1969) *Clin. exp. Immun.*, **5**, 409.
SOLOWEY, A. C. AND RAPAPORT, F. T.—(1965) *Surgery Gynec. Obstet.*, **121**, 756.
SOUTHAM, C. M.—(1968) *Cancer Res.*, **28**, 1433.
SOUTHAM, C. M., BRUNSCHWIG, A. AND DIXON, Q.—(1962) in ' Biological Interactions in Normal and Neoplastic Growth '. Edited by M. J. Brennan and W. L. Simpson. Boston (Little, Brown & Co.), p. 723.
THOMSON, D. M. P., KRUPEY, J., FREEDMAN, S. O. AND GOLD, P.—(1970) *Proc. natn. Acad. Sci., U.S.A.*, **64**, 161.
WISTAR, R.—(1968) *Aust. J. exp. Biol. med. Sci.*, **46**, 769.